# Detection, Mapping, and Quantification of Single Walled Carbon Nanotubes in Histological Specimens with Photoacoustic Microscopy

**DOI:** 10.1371/journal.pone.0035064

**Published:** 2012-04-09

**Authors:** Pramod K. Avti, Song Hu, Christopher Favazza, Antonios G. Mikos, John A. Jansen, Kenneth R. Shroyer, Lihong V. Wang, Balaji Sitharaman

**Affiliations:** 1 Department of Biomedical Engineering, State University of New York at Stony Brook, Stony Brook, New York, United States of America; 2 Optical Imaging Laboratory, Department of Biomedical Engineering, Washington University in St. Louis, Saint Louis, Missouri, United States of America; 3 Department of Bioengineering, Rice University, Houston, Texas, United States of America; 4 Department of Pathology, School of Medicine, Stony Brook University Medical Center, Stony Brook, New York, United States of America; 5 Department of Periodontology and Biomaterials, Radboud University Nijmegen Medical Center, Nijmegen, The Netherlands; University of California Merced, United States of America

## Abstract

**Aims:**

In the present study, the efficacy of multi-scale photoacoustic microscopy (PAM) was investigated to detect, map, and quantify trace amounts [nanograms (ng) to micrograms (µg)] of SWCNTs in a variety of histological tissue specimens consisting of cancer and benign tissue biopsies (histological specimens from implanted tissue engineering scaffolds).

**Materials and Methods:**

Optical-resolution (OR) and acoustic-resolution (AR) - Photoacoustic microscopy (PAM) was employed to detect, map and quantify the SWCNTs in a variety of tissue histological specimens and compared with other optical techniques (bright-field optical microscopy, Raman microscopy, near infrared (NIR) fluorescence microscopy).

**Results:**

Both optical-resolution and acoustic-resolution PAM, allow the detection and quantification of SWCNTs in histological specimens with scalable spatial resolution and depth penetration. The noise-equivalent detection sensitivity to SWCNTs in the specimens was calculated to be as low as ∼7 pg. Image processing analysis further allowed the mapping, distribution, and quantification of the SWCNTs in the histological sections.

**Conclusions:**

The results demonstrate the potential of PAM as a promising imaging technique to detect, map, and quantify SWCNTs in histological specimens, and could complement the capabilities of current optical and electron microscopy techniques in the analysis of histological specimens containing SWCNTs.

## Introduction

Single walled carbon nanotubes (SWCNTs) have emerged as one of the most widely-researched nanomaterial for *in vivo* bio-imaging, drug delivery, and tissue engineering applications [1]–[3]. Their suitability for these *in vivo* applications is currently being tested in small animals, where post-mortem histological analysis is the most widely used technique to evaluate the accumulation of SWCNTs in the microscopic anatomy of cells and tissues [4]. However, detection, mapping, and quantification of SWCNTs in histological specimens (thin films 1–20 µm thick) still remain a challenge. To date, electron and optical microscopy-based techniques allow excellent detection of SWCNTs in histological specimens; however, quantifying the anatomical distribution of SWCNTs in these specimens still remains a challenge (Table 1) [4]–[6]. The image contrast of transmission electron microscopy under conditions typically used for biological specimens does not allow for accurate quantification of trace amounts (ng–µg) of SWCNT accumulation in cells and tissues, nor does it permit clear differentiation between SWCNTs, and dense cellular components (such as ribosomes) [7]. Raman microscopy of histological samples requires spectroscopic measurements to detect resonant scattering peaks, and would require laser sources with multiple wavelengths that allow tuning to these resonant peaks [8]. Additionally, SWCNTs synthesized by various processes are typically comprised of a mixture of multiple species (different chiralities, semiconducting or metallic), which impedes accurate quantification because the Raman cross sections for these SWCNTs species are currently not available from experimental studies [9]. Near infrared fluorescence microscopy can only detect individual semiconducting SWCNTs, cannot detect metallic SWCNTs, and allows reduced or no detection of SWCNT aggregates. Thus, unless the histological specimens mainly comprise of individual semiconducting SWCNTs, quantification would be difficult [10]. Bright field optical microscopy of dye stained histological specimens has limitations as well [5]. It is difficult to distinguish SWCNTs from the intracellular organelles especially near the nuclear region as well as debris and microbubbles formed during the sectioning process because they appear similar to SWCNTs [5]. Recently, photoacoustic microscopy (PAM) has been demonstrated to detect SWCNTs with high spatial resolution and sensitivity [11]–[14]. PAM's excellent sensitivity stems from the strong optical absorption properties of SWCNTs, which constitutes the primary contrast mechanism in PAM. In this work, we have investigated the efficacy of PAM to detect, map, and quantify the distribution of SWCNTs in a variety of histological specimens.

**Table 1:** The limitations of transmission electron microscopy, Raman spectroscopy, near-IR fluorescence microscopy, and bright-field optical microscopy and the potential advantages of photoacoustic microscopy in detection, mapping, and quantification of SWCNTs in histological specimens.

#Limitations	Advantages			
Transmission electron microscopy	Raman microscopy	Near IR fluorescence microscopy	Bright-field optical microscopy	Photoacoustic Microscopy
**Detection of SWCNT in histological specimens**				
(a) Image formed by partial absorption of an electron beam transmitted through the specimen.(b) Allows detections. Sometimes difficult to discriminate between SWCNT and carbon-rich cell structures due to low imaging contrast (7,30).	(a) Image formed by inelastic or Raman scattering of light by the specimen.(b) Allows detection. Weak scattering signal requires high laser power, may not preserve the sample for multiple analysis (8).(c) Need use of near-resonant laser sources. Thus, laser sources that can be tuned to multiple wavelengths would be needed (8–9)	(a) Image formed by NIR fluorescent emission of the specimen.(b) Allows detection. Low intensity and aggregation limits detection (11).(c) Quantum yields are low and dependent on chemical environment such as pH, concentration and salinity (31).(d) Physical adsorption of biomolecules like proteins and DNA quenches the NIR photoluminescence (22–24).	(a) Image formed by partial attenuation of white-light illumination transmitted through the specimen(b) Allows detection. Carbon nanotubes appear as black particles, difficult to distinguish between fine air bubbles, dust impurities, and dense stains which also appear black [5].	(a) Image formed by optical absorption-induced ultrasound emission of the specimen(b) Allows detection. Aggregation of SWCNTs does not reduce photoacoustic signal intensity.(c) No quenching of signals observed over long exposures.
**Quantification of SWCNT in histological specimens**				
(a) Only qualitative information obtained (7).(b) Has not been demonstrated in thin film histological specimens.	(a) SWCNTs synthesized by various processes are a mixture of semiconducting and metallic with characteristic resonance scattering peaks. Thus, quantifying them in a mixture is a challenge (9).(b) Has been demonstrated in SWCNT biodistribution studies when biological specimens are prepared as fluid solutions (9).(c) Has not been demonstrated in SWCNT biodistribution studies when biological specimens are prepared as thin film histological specimens.	(a) Accurate quantification is difficult because of the low signal intensity. Signals obtained only by individual semi-conducting SWCNTs which unless enriched typically represent only 1/3 of SWCNT sample.(b) Aggregates quench the NIR fluorescence, and hence challenging to quantify (10).(c) Has not been demonstrated in thin film histological specimens.	(a) Quantification of SWCNTs not possible due to reason listed above.(b) Has not been demonstrated in histological specimens.	(a) Quantification of SWCNTs in histological specimens possible.(b) Has not been demonstrated in histological specimens.
**Mapping of SWCNT in histological specimens**				
(a) Accurate mapping not possible.(b) No reports available on thin film histological specimens	(a) Mapping possible, but could be challenging.(b) No reports available on thin film histological specimens	(a) Accurate mapping not possible unless sample contains only individual semiconducting SWCNTs.(b) No reports available on thin film histological specimens	(a) Accurate mapping not possible.(b) No reports available on thin film histological specimens	(a) Mapping possible.(b) No reports available on thin film histological specimens

## Results

**Figure 1:**
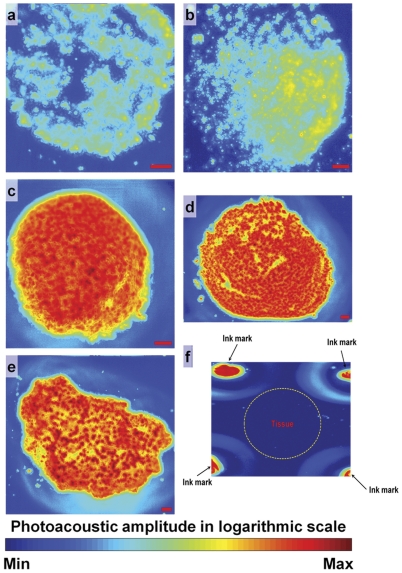
Acoustic-resolution photoacoustic microscopy (AR-PAM) of single walled carbon nanotubes (SWCNTs). PAM images at 4 different concentrations (a) 400 ng, (b) 800 ng, (c) 10 µg and (d) 40 µg of the SWCNTs spread across a given area on a glass slide, (e) an unknown concentration of SWCNTs spread on the surface of a human breast cancer tissue section and (f) unstained human breast cancer tissue. All scale bars are 1 mm.

**Figure 2:**
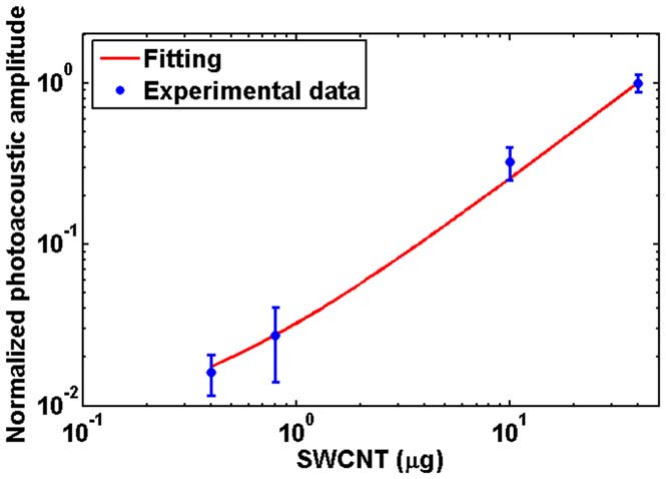
The linear-fitted standard curve of photoacoustic signals obtained from 400 ng, 800 ng, 10 µg and 40 µg of single walled carbon nanotubes. For each concentration, 3 spots were spotted in a small area on a glass slide and air dried. The photoacoustic amplitude of the nanotubes distributed in each spot is integrated and represents the total photoacoustic amplitude. Each data point in Fig. 2 represents the average photoacoustic amplitude of 3 spots at each concentration, and the error bar represents the standard deviation. The R^2^ value for the linear-fitting curve is 0.992.

To generate the standard curve for SWCNT quantification, different concentrations (400 ng–40 µg) of SWCNTs spread on glass slides were imaged using AR-PAM (Figs. 1a–1d). The total photoacoustic signal of SWCNTs at each concentration was averaged from 3 spotted areas (Table 2). The standard curve shows a linear dependence (r^2^ = 0.992) between the total photoacoustic signal and the SWCNT concentration (Fig. 2), which can be used to estimate the SWCNT mass in unknown tissue samples. Since AR-PAM and OR-PAM have similar detection sensitivities, which has been validated in blood vessel imaging [11], [17], we did not repeat the same calibration process for OR-PAM. Figure 1e shows the AR-PAM image of an unstained histological section of breast cancer tissue. Using the standard curve, the SWCNT mass is estimated to be 31.1 µg, which is very close to the actual mass of 30 µg spread on the breast cancer sample.

**Table 2:** Concentration-dependent photoacoustic signals produced by single walled carbon nanotubes (SWCNTs).

Concentration	Average total photoacoustic signal (×10^4^)	Standard deviation (×10^4^)	Average Noise level per resolution voxel	Noise-equivalent mass sensitivity
400 ng	1.94	0.55	0.29	6.0 pg
800 ng	3.30	1.60	0.22	5.3 pg
10 µg	39.00	9.10	0.30	7.7 pg
40 µg	120.50	14.66	0.26	8.6 pg

For each concentration, 3 spots were spotted. In each spot, the photoacoustic signal signals of all the trace amounts of SWCNTs are integrated and represented as the total photoacoustic signal. Then the average photoacoustic signal and the standard deviation were obtained from the three spots of each concentration. The noise-equivalent mass sensitivity was calculated based on the quantification of the average noise level per resolution voxel. The photoacoustic signals of all the above concentrations were used to generate the standard curve in Fig. 2.

The noise-equivalent detection sensitivity of PAM was further estimated, which is defined as the SWCNT mass per AR-PAM resolution voxel (45×45×15 µm^3^) that can generate a photoacoustic signal equal to the system noise level. As shown in Table 2, the noise-equivalent mass sensitivity is ∼7 pg, translating to a detection sensitivity of ∼0.23 fg/µm^3^. Photoacoustic signals from breast cancer tissues with no SWCNTs (Fig. 1f, highlighted with a dashed yellow circle) were measured to be very close to the average noise level of the SWCNT samples shown in Figs. 1a–1d, suggesting a similar noise-equivalent mass sensitivity of 7 pg *in vivo*. Usually, a signal-to-noise ratio of at least 2 is required for imaging and sensing, which corresponds to a practical sensitivity of 14 pg. In contrast to the weak signal of the native cancer tissue, four fiducial ink marks that delineate the tissue boundaries show relatively strong photoacoustic signals (Fig. 1f, black arrows).

**Figure 3:**
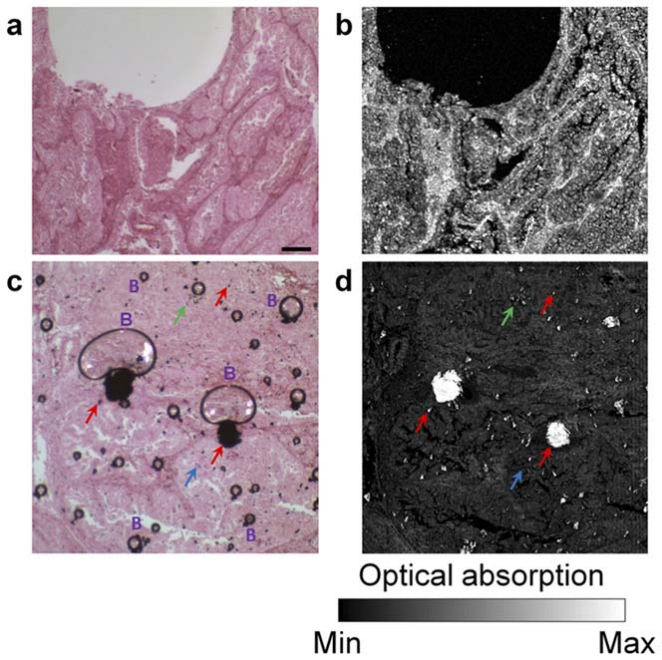
Comparison between conventional transmission-mode optical microscopy and optical-resolution photoacoustic microscopy (OR-PAM) of human breast cancer tissues in the absence and presence of single walled carbon nanotubes (SWCNTs). Optical images of H&E stained breast cancer tissue sections without (a) and with (c) SWCNTs. OR-PAM images of the same human breast cancer tissue without (b) and with (d) SWCNTs. In panel c, artifacts generated during the preparation of histological specimens such as micro- and nano-sized bubbles (represented by the letters B) nuclear stains (green arrows) and, dust particles (blue arrows) are shown and appear black along with SWCNTs aggregates (red arrows). In panel d, only the SWCNT aggregates are detected (red arrows) and the artifacts are not detected.. The scale bar in (a) is 200 µm and applies to all the panels.

To demonstrate the high sensitivity and specificity of PAM for SWCNT detection, OR-PAM and transmission-mode optical microscopy images of H&E stained human breast cancer sections with and without SWCNTs, respectively were compared (Fig. 3). In the absence of SWCNTs, H&E stains show similar imaging contrasts in transmission-mode optical microscopy (Fig. 3a) and OR-PAM images (Fig. 3b). However, in the presence of SWCNTs (Figs. 3c, red arrows) as well as the artifacts created during specimen preparation such as trace impurities, excess nuclear stains (Figs. 3c, green arrows), dust (Figs. 3c, blue arrows), and small bubbles (Figs. 3c, represented as B), the transmission-mode optical microscope has difficulty in distinguishing the SWCNTs from these dark-spot-like artifacts. In contrast, OR-PAM can clearly differentiate SWCNTs (Figs. 3d, red arrows) from the aforementioned artifacts (Figs. 3d, green and blue arrows) with a more than 14 dB difference in photoacoustic signal strength. Note that the photoacoustic signal of H&E stains is also much weaker compared to that of SWCNTs (Fig. 3d). The presence of SWCNT aggregates Figs. 3c and 3d was confirmed by Raman spectroscopy.

**Figure 4:**
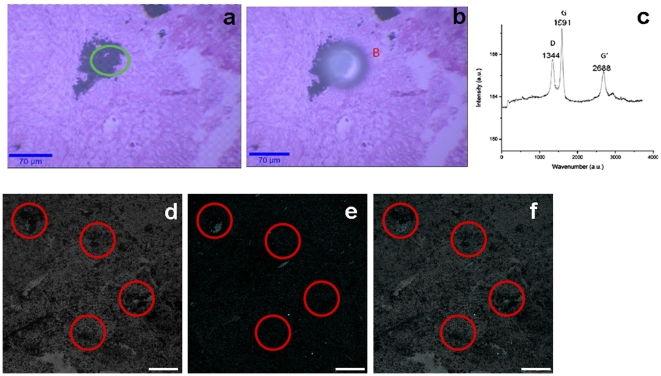
Confocal Raman (a–c) and NIR luminescence (d–f) imaging of stained and unstained breast cancer tissue with SWCNTs. Optical transmission microscopy before (a) and after (b) laser excitation during Raman imaging of SWCNTs in H&E stained breast cancer tissue. (c) Raman spectrum obtained on the same tissue using a WITec Alpha 300 Confocal Raman Microscope excited with a 532 nm laser. The laser intensity used damages the tissue under investigation and creates artifacts represented by letter ‘B’. The Raman spectra of SWCNTs showing G (tangential oscillations of carbon atoms along the nanotube axis) and D (vibrational modes of disordered carbon) bands. (d) Confocal images of breast cancer tissue containing SWCNTs in the bright field. (e) NIR Confocal fluorescence image of the tissue with SWCNTs (λex = 650 nm, λem = 750–900 nm. (f) Overlay of optical and NIR images of breast cancer tissue with SWCNT. Circles show the SWCNT aggregates.

Raman spectroscopy (Figs. 4a–4c) and NIR fluorescence (Figs. 4d–4e) also allowed detection of SWCNTs in breast cancer tissues. However, detection of all the SWCNT species, and thus accurate quantification of SWCNTs in the histological samples could not be achieved. Figures 4a and 4b show bright-field optical microscopy images of a cancer tissue section before and after performing Raman spectroscopy (excitation laser wavelength: 532 nm). The characteristic Raman spectra with the G and D bands confirm the presence of SWCNTs (Fig. 4c). However, prolonged exposure of the laser to obtain a good Raman signal generated bubbles and/or ablated the tissues (Figure 4b) limiting their use for continuous or repetitive studies. Figures 4d and 4e show the bright-field, and NIR fluorescence images of breast cancer tissues with SWCNTs, respectively. The bright-field optical microscopy image shows SWCNT aggregates all over the tissue specimen (Fig. 4d, some representative regions are highlighted with the red circles). The NIR fluorescence (excitation: 650 nm; emission: 750–900 nm) microscope could not detect all the aggregated SWCNTs, since the formation of aggregates/clusters decreases photoluminescence signals (Fig. 4e). The overlay of the bright field and fluorescence images (Fig. 4f) show weak or no fluorescence signals in portions of the specimen that contain SWCNT aggregates (red circled regions).

**Figure 5:**
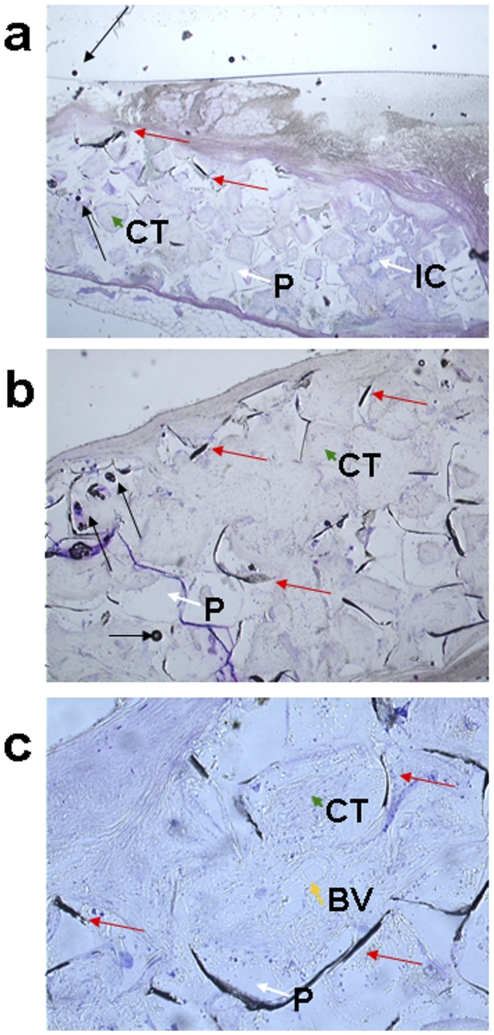
Histological sections of poly(propylene fumarate) (PPF) scaffold implanted rabbit subcutaneous tissue stained with methylene blue. Images are presented at 2× (a), 4× (b) and 10× (c) magnification. The connective tissue (CT) and PPF scaffold (P) is present in all the sections. Inflammatory cells (IC) and blood vessel (BV) are also observed in the images (a) and (c). The red arrows in all the images represent regions of intense staining and the black arrows represent air bubbles and stain spots.

**Figure 6:**
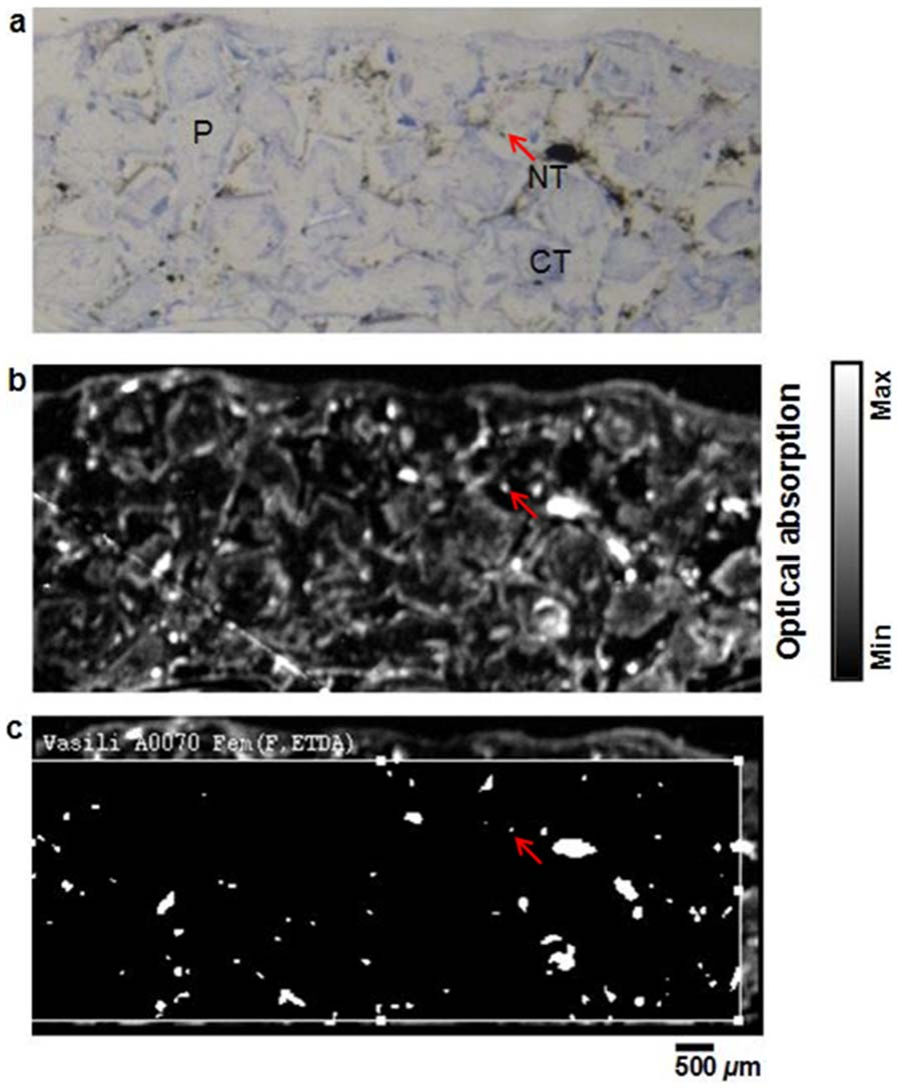
Comparison between conventional transmission-mode optical microscopy and optical-resolution photoacoustic microscopy (OR-PAM) of subcutaneous rabbit tissue implanted with poly(propylene fumarate) (PPF) scaffold for 12 weeks and labeled with SWCNTs. (a) The optical image and (b) the OR-PAM image of methylene blue and basic fuchsin stained subcutaneous rabbit tissue with SWCNTs. (c) Image processed using the SCANCO software by subtracting the background signal of the breast tissue. The SWCNTs appear as black aggregates (NT), the blue stained areas represent the connective tissue CT, and ‘P’ represents the PPF scaffold in the optical images.

Taking advantage of PAM's high sensitivity and specificity to SWCNTs, we further evaluated its efficacy for mapping SWCNTs in histological specimens, where traditional bright-field optical microscopy has poor specificity. For example, in the bright-field optical microscopic images of a methylene blue stained rabbit subcutaneous tissue specimen at varying magnifications (Fig. 5a: 2×, Fig. 5b: 4×, and Fig. 5c: 10×), SWCNT aggregates (green arrows), bubbles (black arrows), and intense stains (red arrows) all appear black and are difficult to differentiate. For comparison, a rabbit subcutaneous tissue specimen with tissue engineering implants was imaged using both bright-field optical microscopy (Fig. 6a) and OR-PAM (Fig. 6b). Polypropylene fumarate polymer (PPF) scaffolds (P) and connective tissues (CT), which show similar morphology to small SWCNT aggregates (NT, red arrow) in Fig. 6a, can be clearly distinguished from SWCNTs by OR-PAM (Fig. 6b). The areas of dye-stained tissues and polymer scaffolds present much weaker photoacoustic signals as compared to the SWCNT areas, and thus can be considered as background. To further isolate SWCNT signals, a method was developed based on SCANCO–IPL to subtract the background signals (Fig. 6c). With background subtraction, we were able to quantify the area of the SWCNT distribution to be 28.7 mm^2^, which accounted for only 2.38% of the total area of the subcutaneous tissue imaged by OR-PAM (1205 mm^2^).

## Discussion

The aim of this study was to investigate the efficacy of PAM to detect, map and quantify SWCNTs in a variety of histological specimens. The histological specimens consisted of cancer and benign tissue biopsies (histological specimens from implanted tissue engineering scaffolds).

The cancer tissue sections were used as tissue phantoms to determine, and validate the capabilities of PAM to detect, and quantify SWCNT. Since these experiments needed a prior knowledge of the concentrations, known amounts of SWCNTs were spotted on the breast cancer histology specimens. Our results indicate that even extremely low amounts of SWCNTs can generate strong photoacoustic signals, providing sufficient contrast to detect and distinguish them from the background tissue. Further, the SWCNTs could be accurately quantified and mapped in the specimens. To quantify the SWCNTs, we prepared a linear standard curve of photoacoustic signal strength as a function of SWCNT concentration, and estimated the noise-equivalent detection sensitivity to be 7 pg. The accuracy of the quantification using the standard curve was validated by performing a single blind study to determine the concentration of SWCNTs present in a breast cancer tissue (Figure 1e). The measurement error was 3.3%, indicating that our method allows accurate quantification of the SWCNTs even at low concentrations.

Subsequently, *in vivo* experiments were conducted with SWCNTs, and postmortem benign histological specimens of explanted tissues were prepared. PAM was performed on these histological tissues to validate the detection, quantification, and mapping of unknown trace amounts of SWCNTs. To achieve these ends, SWCNT-incorporated tissue engineering scaffolds implanted subcutaneously in rabbits over a period of 4 weeks were explanted en-bloc post-mortem with the surrounding tissues, and tissue histological sections were prepared. These tissue sections were imaged with bright-field optical microscopy and PAM. Image analysis was further used to map, and quantify the SWCNT distribution in tissue specimens. The bright field optical microscopy showed large aggregates of SWCNTs in subcutaneous tissues; however, small aggregates could hardly be distinguished from dye stains, and polymer scaffolds (Fig. 6a). In contrast, OR-PAM showed a significant difference in optical absorption between SWCNTs, and the background (dye stains and polymer scaffolds) (Fig. 6b). A user-defined grey threshold similar to ones used in histomorphometric analysis allowed effective background subtraction [1]. The final processed image showed the mapping of the fine distribution of the SWCNTs, and thereby enables estimation of the SWCNT amounts (Fig. 6c). Thus, PAM along with the developed image analysis protocol should provide greater insight in investigating host responses to SWCNT-based devices and implants [5].

Bright-field optical microscopy (Fig. 3a, 3c, and Fig. 5), Raman spectroscopy (Fig. 4a–4c), and NIR fluorescence microscopy (Fig. 4d–4f) of histological specimens containing SWCNTs were performed, and compared with OR-PAM. Bright-field optical microscopy of dye-stained histological specimens containing SWCNTs is by far the most widely used technique. The results of this study, and by others [5] clearly show that the capabilities of this technique are limited by artifacts created during the specimen preparation, including impurities, false or dense staining of colored dyes or organic fluorophores, and microbubbles. Generally, these artifacts appear black, and are difficult to distinguish from SWCNTs (Fig. 3c). Though recent progress in quantifying SWCNTs with Raman scattering has been achieved, it is limited by a weak signal to noise ratio and the requirement of using near-resonant laser sources [8]–[9]. Our experimental results show that use of laser at the intensity needed to obtain a robust Raman signal can generate artifacts (bubbles) in the tissue specimens and/or damage (e.g. ablate) the tissue under investigation (Fig. 4c). Thus, this technique may not be useful for multiple analyses of the tissue specimens. NIR fluorescence microscopy, another widely used technique for SWCNT analysis [10], could not be used for the quantification or mapping of SWCNTs in thin film histological specimens (Fig. 4d–4f). The results indicate that the formation of aggregates/clusters decreases the photoluminescence signal. Additionally, the metallic SWCNTs show no photoluminescent signal, and photo-bleaching occurred over prolonged exposures. Furthermore, it has been shown recently that adsorption of biomolecules such as proteins and DNA quench the NIR photoluminescence [19]–[21]. Thus, even though NIR fluorescence microscopy could be suitable for the detection, quantification, and mapping of individual semiconducting SWCNTs in histological specimens, it may not be an ideal tool for quantitative analysis when SWCNTs aggregates or metallic SWCNTs are also present in histological specimens [11]. PAM holds the potential to complement the capabilities of the other optical imaging-based techniques. The large optical absorption coefficient, and wide absorption spectrum of SWCNTs allows highly specific photoacoustic detection at low laser exposure levels, and a variety of optical wavelengths (Fig. 3d) [22]. Furthermore, the detection sensitivity of PAM (down to picogram range) is similar, or better than existing optical techniques such as NIR fluorescence microscopy, and Raman spectroscopy [10], [23]–[25]. The deep tissue penetration of AR-PAM also shows the potential for *in vivo* applications.

### Conclusion

In the present study, we have validated and demonstrated the efficacy of PAM to detect, map and quantify trace amounts of SWCNTs in a variety of histological tissue specimens. The detection sensitivity of PAM to SWCNTs could reach down to picogram levels. Image processing analysis similar to that used in micro-computed x-ray tomography further enables mapping of SWCNT distribution in a rabbit subcutaneous tissue specimen with tissue engineering scaffolds and thereby identifying tissue regions containing trace amounts of SWCNTs. Taking advantage of the high optical absorption contrast of SWCNTs, PAM could provide complementary information to existing electron and optical microscopy techniques in the histological analysis of tissue sections containing SWCNTs. PAM is highly sensitive to SWCNTs and possesses scalable penetration/resolution, which promotes its utility in numerous future applications in detection, mapping, and quantification of SWCNTs *in vivo*.

## Materials and Methods

### Ethical review of procedures

All experimental animal and human procedures were carried out in conformity with the guidelines of the US National Institutes of Health. The laboratory animal protocols for this work were approved by University of Nijmegen's Animal Care and Use Committee. De-identified archival formalin-fixed paraffin-embedded remnant tissue blocks were processed from the archival collections of the Department of Pathology at Stony Brook University Medical Center. These materials do not meet the Department of Health and Human Services Office for Human Research Protections definitions for human tissue research.

### Standard curve for quantification of SWCNT concentration

Commercially available as-received SWCNTs (#519316, Sigma-Aldrich, St. Louis, MO, USA) were used for this study. The average SWCNT diameter was 1.4 nm. To measure the standard curve, SWCNTs were dispersed at a concentration of 1 mg/ml of 1% pluronic F-127 and sonicated at 25 W for one hour using a probe sonicator. After sonication, SWCNTs with concentrations of 400 ng, 800 ng, 10 µg, and 40 µg were individually spotted in a small area on the glass slide and air dried. For each concentration, 3 areas were spotted and imaged with acoustic-resolution PAM (AR-PAM). The average total photoacoustic signal from the 3 spots for each concentration was obtained, the standard deviation was calculated, and the standard curve delineating the relationship between photoacoustic signal strength and SWCNT concentration was generated.

### Cancer tissue histological procedures

Human breast cancer tissue sections were cut 4 µm thick for examination. A single blind study was designed to determine the concentration of SWCNTs in the cancer tissue sections using AR-PAM. SWCNTs of 30 µg were spotted on the cancer tissues and air dried. The amount of the spotted SWCNTs was unknown to the investigators performing the photoacoustic measurements. The total photoacoustic signal generated from the unknown samples was measured, and the SWCNT mass quantity was extrapolated from the standard curve.

To demonstrate the high sensitivity and specificity of PAM to detect and quantify SWCNTs, cancer tissues with and without the SWCNTs were stained with hematoxylin and eosin (H&E) and examined by both optical-resolution PAM (OR-PAM) and an Olympus microscope (BX-51).

### SWCNT-tissue engineering scaffolds

Porous (84% volume porosity) tissue scaffolds consisting of SWCNTs dispersed (0.5 wt%) in a biodegradable, biocompatible polymer poly(propylene fumarate) (PPF) were used. The description of the preparation and characterization of these tissue engineering scaffolds, their subcutaneous implantation and retrieval in rabbits is described in detail elsewhere [15], [5]. Briefly, the implants and surrounding tissues, retrieved *en bloc*, were cut into smaller samples. Following fixation in a 10% buffered formalin solution; the samples were then prepared for histological analysis. They were dehydrated in a graded series of ethanol, and then embedded with methylmethacrylate, which was subsequently polymerized. Transverse sections of 10–15 µm were made using a modified diamond blade microtome (Leica Microsystems SP1600, Rijswijk, The Netherlands), with three sections obtained from each implant. These sections were stained with methylene blue for evaluation by light microscopy (Leica DMLB, Rijswijk, Netherlands).

### Photoacoustic Microscopy

In PAM, the biological tissue is irradiated by a focused short-pulsed laser beam (570 nm in the present study) and the acoustic pulse induced as a result of transient thermoelastic expansion of the tissue is detected by a focused ultrasonic transducer to form an image. To maximize imaging sensitivity, the optical illumination and the ultrasonic detection are configured coaxially and confocally in our PAM systems. Thus, the sensitivity of PAM is determined by the overlapping of the dual foci [16], [19]. Two-dimensional raster scanning of the overlapped optical and acoustic foci, in combination with time-resolved ultrasonic detection, reveals the volumetric distribution of endogenous or exogenous optical absorbers (SWCNTs in this case). Diffraction-limited optical focus (10 times finer than the acoustic focus) can be used to achieve micron-level lateral resolution within the quasiballistic regime in biological tissues (∼1 mm deep into tissue). This technology is referred to as OR-PAM. By contrast, tight acoustic focusing, in combination with weak optical focusing, is employed by AR-PAM to achieve 50-µm lateral resolution in the optical diffusive regime (∼3 mm deep into tissue). Both OR-PAM and AR-PAM were tested in the present study, predicting the potential of multiscale detection, mapping, and quantification of SWCNTs *in* vivo [17], [18]. Laser pulse energies of 1 mJ (AR-PAM) and 40 nJ (OR-PAM) were used to image the samples. Detailed system descriptions can be referred to our previous publications [11], [13], [17].

### Raman Spectroscopy

Raman spectroscopy was performed using the WITec Alpha (WITec Instruments Corp., Maryville, TN) at 532 nm laser excitation at room temperature. The human breast cancer tissue sections containing SWCNTs were placed under a confocal microscope with the laser coaxially attached with the Raman detector. The laser energy used for Raman spectroscopy was 2400 mJ (green laser power output 60% of 40 mW = 24 mW; exposure time = 100 s, energy = 24 mW×100 s = 2400 mJ).

### Confocal Microscopy

Confocal NIR fluorescence microscopy of breast cancer tissue containing SWCNTs was carried out using Leica SP5 Multiphoton Laser scanning Microscope (Broadband MaiTai laser, excitation laser wavelength = 650 nm, 63X/1.4 oil immersion objective).

### Image processing

SCANCO image processing language (IPL) software (SCANCO Medical AG, Switzerland), which has been widely used to analyze computed tomography (CT) images of bone tissues, was employed to process the SWCNT images obtained by PAM. Initially, two-dimensional Gaussian filters with sigma of 0.5 and support of 1 were applied to smooth the image and to remove noise. The filtered image was then segmented with a global threshold of 30% of the maximum signal. Finally, the background was subtracted from the image to extract SWCNT photoacoustic signals. Inbuilt SCANCO algorithms for quantification of structural information (such as cortical bone thickness, trabecular bone separation, and trabecular number) were used to estimate the distribution of SWCNTs. To evaluate the amount of SWCNTs spread into the areas of interest of different tissues, we quantified the fraction of tissue area where nanotubes are present.
